# Food Production and Consumption in Ordos of Inner Mongolia

**DOI:** 10.3390/foods12051066

**Published:** 2023-03-02

**Authors:** Yexuan Liu, Lin Zhen, Yunfeng Hu

**Affiliations:** 1 Institute of Geographic Sciences and Natural Resources Research, Chinese Academy of Sciences, Beijing 100101, China; 2 School of Resource and Environment, University of Chinese Academy of Sciences, Beijing 100049, China

**Keywords:** food production and consumption, supply and demand, food self-sufficiency, local dependence, Ordos

## Abstract

Ordos is an ecological fragile area in the upstream and midstream of the Yellow River and a component of the ecological security barrier on the northern frontier of China. With population growth in recent years, the contradiction between human beings and land resources has become increasingly prominent, leading to increased food security risks. Since 2000, the local government has implemented a series of ecological projects to guide farmers and herdsmen to transform from extensive production to intensive production, which has optimized the pattern of food production and consumption. It is necessary to study the balance between food supply and demand to evaluate food self-sufficiency. Here, we used the panel data from 2000 to 2020 collected based on random sampling surveys to reveal the characteristics of food production and consumption, the changes in food self-sufficiency rate and the dependence of food consumption on local production in Ordos. The results showed that food production and consumption dominated by grains have been increasing. The residents’ diets were characterized by excessive consumption of grains and meat, and insufficient consumption of vegetables, fruits, and dairy foods. On the whole, the locality has achieved self-sufficiency, because the food supply exceeded the demand during the two decades. However, the self-sufficiency of different food types varied greatly, as some foods, such as wheat, rice, pork, poultry, and eggs, have not been self-sufficient. Due to the increased and diversified food demand of residents, food consumption became less dependent on local production and more dependent on food imported from the central and eastern China, which threatened local food security. The study can provide a scientific basis for decision-makers for the structural adjustment of agricultural and animal husbandry and the structural adjustment of food consumption, to ensure food security and sustainable utilization of land resources.

## 1. Introduction

**Figure 1:**
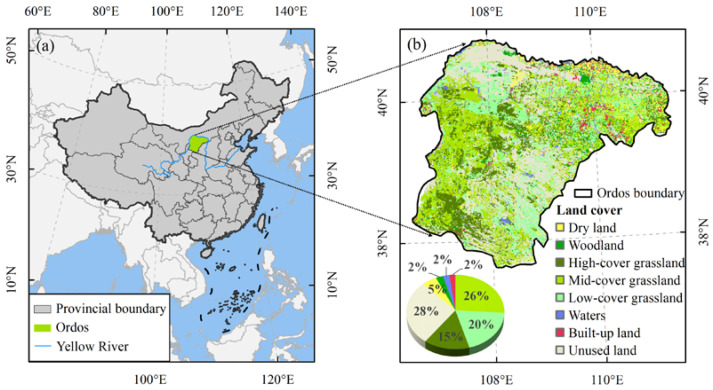
Location (**a**) and land cover (**b**) of Ordos.

The balance of food production and consumption is the key to meeting human food needs and ensuring food security, and also reflects the relationship between human and natural ecosystems. In the past few decades, China has made remarkable achievements in improving agricultural productivity and reducing hunger and malnutrition [1]. The incidence of food shortage has dropped from 10% in 2000 to less than 2.5% in 2020 [1,2]. Although the food supply at the national level has improved [3], food shortages still exist in mountainous areas and pastoral areas lacking cultivated land [4]. Ordos is located in an ecologically fragile zone transitioning from a semi-arid to arid area [5,6], with 90.76% desertified land and only 5% cultivated land (Figure 1), which cannot provide adequate food for local residents. With the growth in population and the improvement in residents’ dietary quality, the contradiction between food production and consumption has become increasingly prominent [7], which brings challenges to the sustainable development of agricultural resources and the ecological environment [8]. At the beginning of the 21st century, the “Natural Forest Protection Project” (2000) and the “Grain for Green Project” (2002) were launched successively in Ordos to reduce the impact of human interference on ecological restoration by delimiting closed protection areas, reducing grazing intensity, and implementing shed feeding of livestock. As an important component of the ecological security barrier on the northern frontier of China, it is necessary to evaluate the food self-sufficiency in Ordos, which can provide a scientific basis for adjusting the structure of agriculture and husbandry, and help to improve the dietary structure of residents in pastoral areas and to predict and cope with food security risks.

Some ecologically fragile areas in China have restored agricultural productivity and improved food self-sufficiency by implementing ecological projects. For example, the grain self-sufficiency of the Loess Plateau could be realized through the “Grain for Green Project”, especially by building terraces and dams, which could raise the grain self-sufficiency rate to 105.25% [9]. It was predicted that the Qinghai Tibet Plateau could also realize grain self-sufficiency, with the self-sufficiency rate exceeding 150%, but the feed grain still could not meet the consumption demand [10]. In addition, the proportion of food consumption expenditure of Chinese residents continued to decline, and the consumption structure was characterized by a decrease in the proportion of grain and edible oil consumption, an increase in the proportion of meat, egg, and dairy consumption, and a faster growth rate for aquatic food expenditure than other foods [11]. There was a gap in the nutrition intake between urban and rural residents. Both urban and rural residents have insufficient protein intake, with the former having an excessive fat intake and the latter having an insufficient fat intake [12]. There is an urgent need to improve the inadequate consumption structure and unbalanced nutrition. From a global perspective, food security is strategically important, but its situation was not optimistic globally. Food supply and demand varied widely in different countries and regions, with the United States, China, Western Europe, and Brazil accounting for 26%, 17%, 11%, and 6% of the global food deficit, respectively [13]. More than half of all countries (120 net food importing countries located in Africa and the Middle East, etc.) depended heavily on food imports to maintain their domestic food supply during 1961–2019 [13]. There was a disjoint between the food production area and consumption area, and the impact of the global food trade on food security increased from 9% to 17% [14], leading to increased instability of the food system [15]. It can be seen that the relevant studies focus on the issues of “food supply and demand”, “food self-sufficiency”, and “food import dependence” at regional, national, and global scales. The common evaluation methods include production and consumption function [16,17], the food nutrition transformation model [18,19], ecological footprint [20,21], life cycle assessment [22,23], food equivalent method [24,25], and the construction of comprehensive index systems [26,27].

Few studies focus on pastoral residents, and their food self-sufficiency and local consumption dependence remain unclear. Most studies have analyzed land productivity [28] and land requirements for food production [29] from the perspective of supply capacity, that is, the independent evaluation of production capacity or consumption capacity separates the relationship between them. In addition, some methods, such as index calculation or model simulation, are difficult to parameterize locally, so the results are uncertain and need to be verified by field survey data. Therefore, based on the city-level statistical data of Ordos from 2000 to 2020, our study combines food production and consumption, and explores whether local production can meet human demand, which are the most important components of human wellbeing. Meanwhile, it can also enrich the food research in the agro-pastoral ecotone and in ecologically fragile areas, where the food security of residents is often not sufficiently addressed. Our goals are to (1) reveal the changes in the structure and quantity of food production and consumption in Ordos; (2) evaluate the food self-sufficiency in Ordos; (3) analyze the dependence of residents’ food consumption on local production in Ordos. This study could raise people’s awareness of the local food production situation and provide guidance for policy making on land use and food production.

## 2. Materials and Methods

### 2.1. Study Area

Ordos is located in the southwest of Inner Mongolia, China (37°35′24″–40°51′40″ N, 106°42′40″–111°27′20″ E), and its north and west are surrounded by the upstream and midstream of the Yellow River (Figure 1a). Ordos has a temperate semi-arid continental climate, and the annual evaporation is about seven times of the annual precipitation (Table 1). Under the influence of a semi-arid climate and human activities, the Mu Us Sandy Land (28.78% of the total land area) and Kubuqi Desert (19.17% of the total land area) were formed in the south and north of Ordos, respectively [30]. The large-scale ecological projects since the beginning of the 21st century have achieved ecological and economic benefits. “The 6th Kubuqi International Desert Forum” and “The 13th Session of the Conference of the Parties to the United Nations Convention to Combat Desertification” were held in Ordos in 2017, and more than 10,000 participants from 196 countries and regions shared their experiences in combating desertification. By 2021, the governance rates of the Mu Us Sandy Land and Kubuqi Desert have reached 70% and 25%, respectively.

Grassland accounts for 61% of the land area of Ordos, including 26% of mid-coverage grassland, 20% of low-cover grassland, and 15% of high-cover grassland (Figure 1b). Cropland accounts for 5%, mainly distributed in the alluvial plain of the Yellow River and Wuding River Basin of Dalad Banner, Hanggin Banner, and Jungar Banner. The proportion of grassland and cropland is 11.8:1. Animal husbandry is the pillar industry of Ordos, and the production and consumption structure of residents is dominated by livestock products [31]. Ordos is located in the gold milk source belt at 40° N and is one of the world’s centers of the cashmere and wool industry. In 2020, the permanent population of Ordos reached 2,155,600, with the population of Han nationality accounting for the majority (89.3%), followed by the Mongolian population (9.7%) (Table 1). The disposable income per capita and consumption expenditure per capita of all Ordos residents are 42,400 yuan and 25,400 yuan, respectively. In rural areas, the per capita income of agriculture and animal husbandry is 11,137 yuan, accounting for 51.6% of the disposable income per capita of rural residents, and Engel’s Coefficient drops to 24.7%.

### 2.2. Data Collection

#### 2.2.1. Statistical Survey Data

The data of crop sown area, crop yield, output of livestock products, permanent population, and food consumption per capita in Ordos were obtained from the statistical yearbook, including *Ordos Statistical Yearbook (2001–2021)*, *Inner Mongolia Autonomous Regional Rural Social Economic Statistical Yearbook (2001–2005)*, *Inner Mongolia Economic and Social Survey Yearbook (2006–2014)* and *Inner Mongolia Survey Yearbook (2015–2021)*. We used the data of food consumption per capita in Yulin, Shaanxi Province, adjacent to Ordos, Inner Mongolia, to replace the missing data.

The data of food origin was derived from the sampling survey of food safety supervision organized by the Ordos Market Supervision Administration from October 2020 to June 2022. The sampling scope covered the wholesale markets of agricultural products, food processing factories, and supermarkets in each banner/district of Ordos. We collected information about the food production area, production date, and the producer and seller of the sampled food, and screened 359 production areas of the main food types.

#### 2.2.2. Household Survey Data

The food consumption data were obtained from the household surveys conducted by the Ordos survey team of the National Bureau of Statistics. The survey team demarcated the survey scope according to the population’s permanent residence, then selected the survey households by a random sampling method and supplemented the municipal household survey samples based on provincial household survey samples. The sample sizes of household surveys were about 300–400 households, involving about 1000 permanent residents (Table 2). The sampling error of survey data was controlled within 1% at the 95% confidence level.

The food consumption data was collected by a household income–expenditure survey and a living condition survey, mainly including the following two aspects of respondent bookkeeping and investigator interview: (1) The respondents recorded their daily food consumption in the diary books formulated by the National Bureau of Statistics, including grains, edible oils, vegetables, meat, eggs, dairy foods, and other food consumed by residents at home. The food consumption of peasant households and pastoral households included not only the food purchased, but also the food they produced for their own use. Investigators checked the bookkeeping and collected the above data monthly. (2) The investigators interviewed household members about the employment of laboring population and household production and investment, and collected these data using quarterly questionnaires. The Ordos survey team encoded, logged, and reviewed the bookkeeping and questionnaires and reported them to the Statistics Bureau, and then the Statistics Bureau conducted data weighting and summarized the data. In addition, our team also conducted a questionnaire survey about the food production and consumption of farmers and herdsmen in Inner Mongolia, which supplemented the survey data of the Ordos survey team (Supplementary Materials).

#### 2.2.3. Spatial Data

The land cover was obtained from the remote sensing monitoring data of China’s land use status in 2020 from the Resource and Environment Science and Data Center (https://www.resdc.cn/data.aspx?DATAID=335, accessed on 10 October 2022). It was based on Landsat TM images and was generated by manual visual interpretation. Land cover types included 6 primary types, namely cropland, woodland, grassland, waters, built-up land, and unused land, and 25 secondary types, including dry land, high-cover grassland, mid-coverage grassland, low-cover grassland, etc.

### 2.3. Classification of Food Types

We referred to the dietary standard of *Dietary Guidelines for Chinese Residents (2022)* and divided food consumption into seven types: grains, tubers, legumes, vegetables, fruits, meat and eggs, and dairy foods. Among them, meat and eggs were further divided into four subtypes: livestock meat, poultry, aquatic foods, and eggs (Table 3).

### 2.4. Calculation of Food Self-Sufficiency

The food self-sufficiency rate is an important measure of food security. It can directly reflect the balance between food production and consumption. Equation (1) is as follows:

(1) $$SSR_{in}=\frac{TFP_{in}}{TFC_{in}}\times 100\%$$

where *SSR* represents the food self-sufficiency rate, *i* represents *i* type of food, *n* represents a certain year, *TFP* represents total food production, and *TFC* represents total food consumption. Equation (2) is as follows:

(2) $$TFC_{in}=FC_{in}\times PRP_{n}$$

where *FC* represents food consumption per capita and *PRP* represents permanent resident population.

## 3. Results

### 3.1. Changes in Food Production and Consumption Characteristics

**Figure 2:**
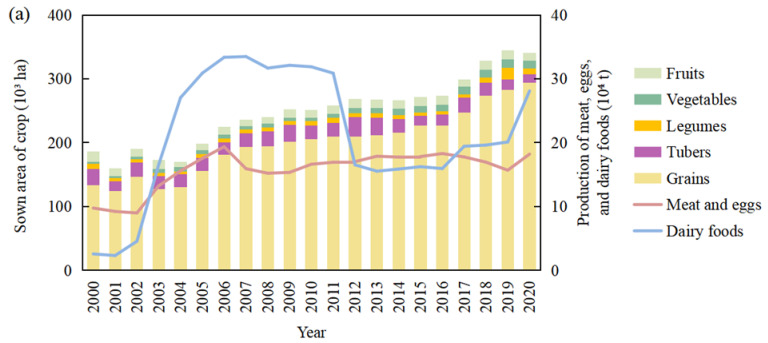

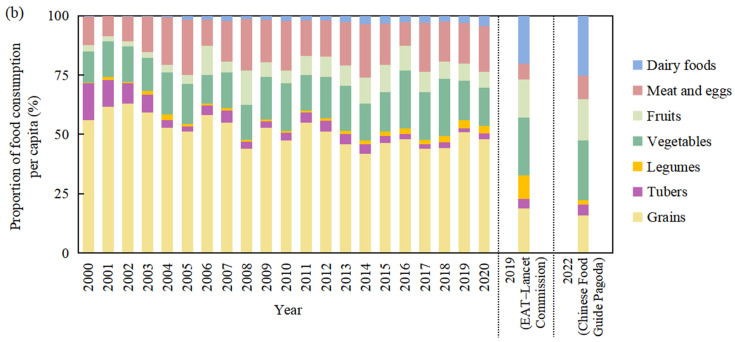
The structural changes in food production (**a**) and consumption (**b**) in Ordos.

The sown areas of grains and tubers in Ordos gradually expanded from 186.26 × 10^3^ ha to 340.36 × 10^3^ ha over two decades (Figure 2a). The growth of sown area was mainly attributed to maize, which was the dominant grain in Ordos, with an area increase of 205.30 × 10^3^ ha. Only the sown area of wheat and potato decreased among all crops. The reason for the decrease in wheat sown area was the increased irrigation and planting costs, such as the purchase of agricultural machinery and production materials, resulting in lower income for farmers. Meanwhile, the expansion of urbanization encroached on cropland suitable for wheat planting. The potato sown area showed a trend of increase first and then decrease, from 14.00 × 10^3^ ha in 2001 to 25.72 × 10^3^ ha in 2009. Since 2010, potato prices have plummeted, reflected by a decline in planting willingness of farmers, and the sown area dropped to 13.28 × 10^3^ ha in 2020.

Compared with the global pattern proposed by the EAT–Lancet Committee (2019) and the Chinese pattern proposed by the Chinese Food Guide Pagoda (2022), the food consumption structure of Ordos residents was unreasonable, and was manifested through excessive consumption of grains and meat and insufficient consumption of vegetables, fruits, and dairy foods (Figure 2b). Grain was the main component of the food consumption of Ordos residents, with the per capita consumption accounting for 51.27%, which has declined slightly in recent years, but it was still more than 30% higher than the global pattern (18.69%) and the Chinese pattern (15.72%). The proportion of meat and egg consumption increased from 11.99% in 2000 to 19.29% in 2020, higher than the 6.77% and 10.06% of the global and Chinese patterns, respectively. The proportion of vegetable and fruit consumption has increased, and the gap between them and the global pattern and Chinese pattern reduced to about 10% in 2020. The consumption of dairy foods has been at a low level of about 6.69 g/day in 2000, lower than the global and Chinese patterns of 250 g/day and 400 g/day, respectively.

**Figure 3:**
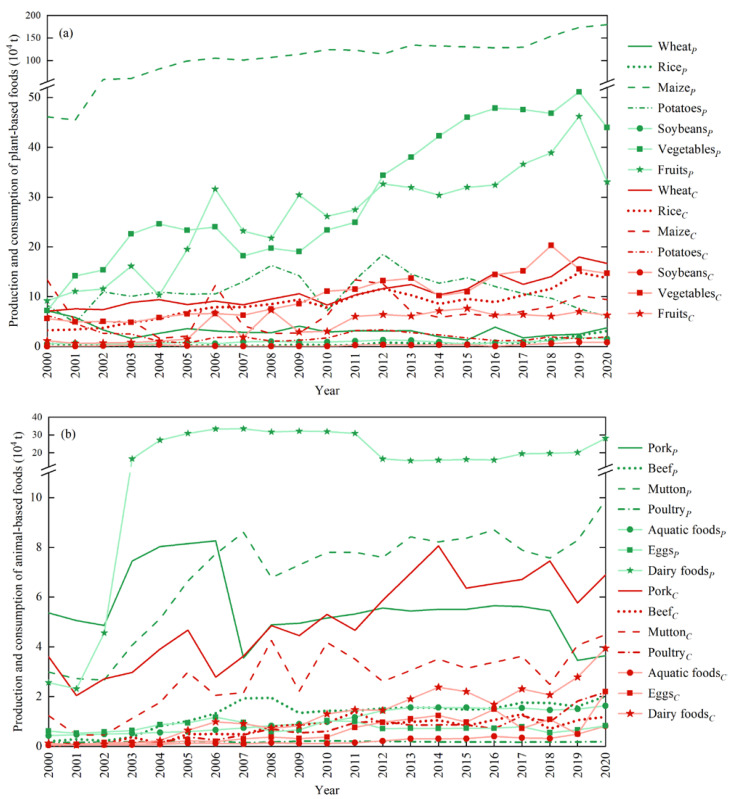
The annual changes in food production and consumption of plant-based foods (**a**) and animal-based foods (**b**) in Ordos (*_C_* and *_P_* represent consumption and production, respectively).

The yields of grains and tubers showed growing trends, which were consistent with the change in sown area. The growth in maize yield was the largest, from 46.12 × 10^4^ t to 180.00 × 10^4^ t in two decades (Figure 3a); the wheat yield decreased from 7.17 × 10^4^ t to 3.74 × 10^4^ t, and the potato yield increased first and then decreased, as from 4.78 × 10^4^ t in 2001 it increased to 14.21 × 10^4^ t in 2009, and then it reduced to 6.02 × 10^4^ t in 2020. Rice was not the main grain in Ordos, with a low yield of 0.17 × 10^4^ t − 0.78 × 10^4^ t before 2017. Since 2017, the yield per unit area of rice increased to about 5250 kg/ha as a result of ameliorating saline–alkali land, with a total yield of 3.11 × 10^4^ t in 2020. In addition, with the diversified demands of residents, the yields of vegetables and fruits also showed relatively large increases: vegetables increased from 7.20 × 10^4^ t to 43.93 × 10^4^ t, and fruits increased from 9.19 × 10^4^ t to 33.04 × 10^4^ t within 20 years, an increase of 6.10 times and 3.60 times, respectively. Mutton production accounted for about 50% of meat production, increasing from 2.99 × 10^4^ t in 2000 to 9.90 × 10^4^ t in 2020, with an increase of 3.31 times (Figure 3b). Especially since 2007, mutton production (8.60 × 10^4^ t) has exceeded pork production (3.55 × 10^4^ t). The production of poultry and eggs was stable, at about 0.18 × 10^4^ t and 0.76 × 10^4^ t, respectively. As part of the gold milk source belt in China, the dairy production in Ordos has grown rapidly from 2.57 × 10^4^ t to 28.11 × 10^4^ t in two decades to supply domestic consumption and export.

The food demand of Ordos residents has been increasing, but the problems of excessive consumption or insufficient consumption of different food types have not been completely solved. Grain consumption per capita was 1.85 times of the global pattern and 1.71 times of the Chinese pattern in 2020. Specifically, the consumption of wheat, rice and maize was 16.67 × 10^4^ t, 13.77 × 10^4^ t, and 9.44 × 10^4^ t, with an increase of 134.80%, 325.57%, and 87.46%, respectively, over the year 2000 (Figure 3a). Vegetables and fruits have reached about half of the global and Chinese patterns, increasing by 9.07 × 10^4^ t (161.74%) and 5.13 × 10^4^ t (443.80%), respectively. Compared with the global pattern, the meat consumption in Ordos was insufficient during 2000–2002 (<70 g/day) and excessive during 2003–2020 (>70 g/day); compared with the Chinese pattern, the meat consumption was insufficient during 2000–2002 (<80 g/day), reasonable during 2003–2012 (80–150 g/day), and excessive during 2013–2020 (>150 g/day). Pork consumption was the highest among meat (8.05 × 10^4^ t; Figure 3b), and the per capita consumption (70.57 g/day) was about 10 times of the global pattern. Influenced by the dietary habits in pastoral areas, residents also consumed a lot of beef and mutton, reaching 2.73 times and 10.53 times the global model, respectively. Beef consumption increased from 0.02 × 10^4^ t to 1.19 × 10^4^ t and mutton consumption increased from 1.23 × 10^4^ t to 4.48 × 10^4^ t during the past 20 years. Poultry consumption increased significantly, reaching 2.18 × 10^4^ t in 2020, about 19.81 times that of 2000. The consumption of aquatic foods was the lowest among types of meat, but it also showed a growth trend, increasing from 0.05 × 10^4^ t to 0.82 × 10^4^ t. The consumption of dairy foods grew rapidly, reaching 3.94 × 10^4^ t in 2020, about 30.31 times that of 2000, but the per capita consumption in 2020 (50.14 g/day) was only 1/5 and 1/8 of the global pattern and Chinese pattern, respectively.

### 3.2. Changes in Food Self-Sufficiency

**Figure 4:**
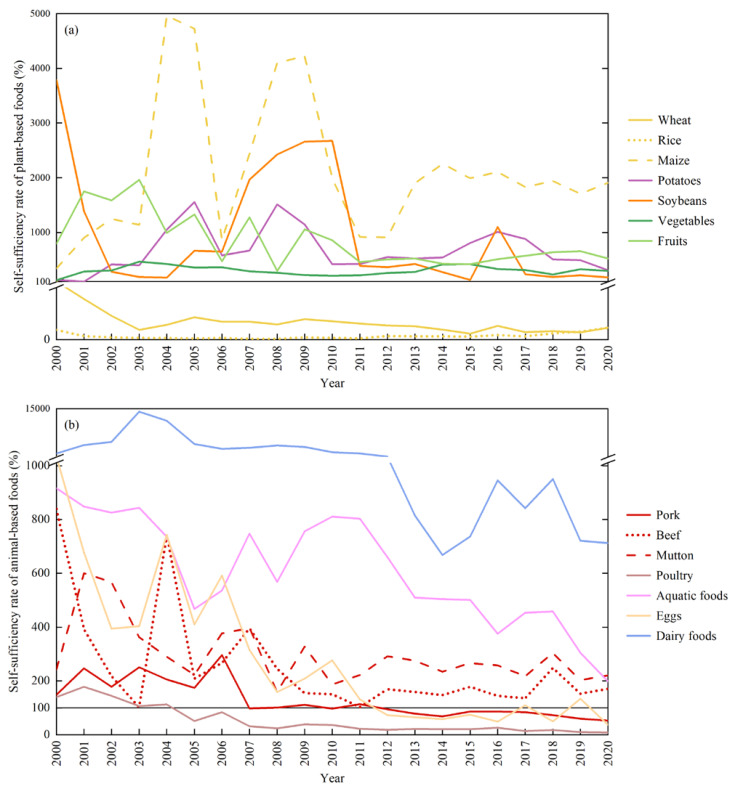
The changes in self-sufficiency of plant-based foods (**a**) and animal-based foods (**b**).

Local foods and foods with less demand have achieved food self-sufficiency, including agricultural and fishery foods, such as maize, potato, soybean, vegetables, fruits, and aquatic products, as well as livestock products, such as beef, mutton, and dairy foods. Maize and potato were local dominant crops, with self-sufficiency rates of 5000% and 1500% respectively (Figure 4a). In addition to meeting food self-sufficiency, they could also be supplied for export, with a commodity rate of more than 80%. All three livestock products, namely mutton, beef and dairy foods, have realized self-sufficiency. In particular, the self-sufficiency rate of dairy foods once reached 14,000% in 2003 and decreased to 712.53% in 2020 (Figure 4b), mainly because the growth rate of production was lower than that of consumption. The self-sufficiency rate of beef decreased from 839.55% to 170.68% over 20 years, while the self-sufficiency rate of mutton has remained at around 300%.

Five types of agricultural foods failed to achieve self-sufficiency: wheat, rice, pork, poultry, and eggs. Wheat and rice have not been self-sufficient for the past two decades, with a demand–supply gap of about 8.02 × 10^4^ t and 8.15 × 10^4^ t, respectively. The self-sufficiency rate of wheat decreased from 77.08% to 22.42% and that of rice increased from 6.61% to 22.59% (Figure 4a). The reasons for the imbalance between supply and demand of wheat and rice were that there were few suitable cropland for planting and the increasing consumption of residents in Ordos. Pork supply was balanced with the consumption demand before 2007, and then the self-sufficiency rate gradually declined to below 100%, so the residents’ consumption could not be satisfied by local production in some years (Figure 4b). Poultry supply was oversupplied in the first five years, and then it changed to a short supply. This was due to the consumption growth of 2.07 × 10^4^ t in two decades, while the production stabilized at 0.18 × 10^4^ t, resulting in the self-sufficiency rate declining to 8.58% in 2020, with a demand–supply gap of 2 × 10^4^ t. The self-sufficiency rate of eggs decreased from 1097.70% to 276.89% in the first decade, but it was still self-sufficient. It then fell to 37.63% in the second decade and was unable to be self-sufficient. It can be seen that the self-sufficiency rates of most foods have shown downward trends regardless of whether they were self-sufficient or not.

**Figure 5:**
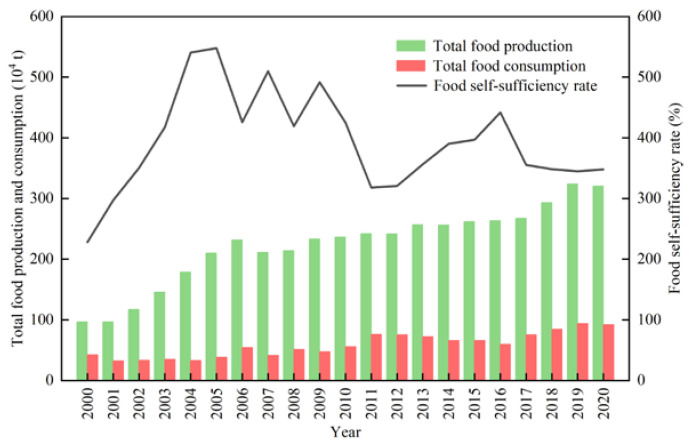
The general trend of food production and consumption.

The total food production could meet the consumption demand in Ordos, and the food self-sufficiency rate was between 228.13% and 547.93%. Both the total production and total consumption of food were on a steady rise, with the total production increasing from 96.58 × 10^4^ t to 320.63 × 10^4^ t and the total consumption increasing from 42.34 × 10^4^ t to 92.09 × 10^4^ t (Figure 5). However, the relationship between production and consumption varied for different food types. The first reason was that with the improvement in residents’ living standards, more types of foods were needed to enrich their dietary structure. Limited by the production conditions, the production capacity of non-local foods could not meet the growing demands of residents, so the scarce foods needed to be transferred from other places. Second, the export of high-quality local foods has changed the relationship of local supply–demand, such as for maize and potato. Therefore, it is necessary to further study the food sources of local residents to determine their dependence on local and non-local food production.

### 3.3. Dependence of Food Consumption on Local Production and Non-Local Production

**Figure 6:**
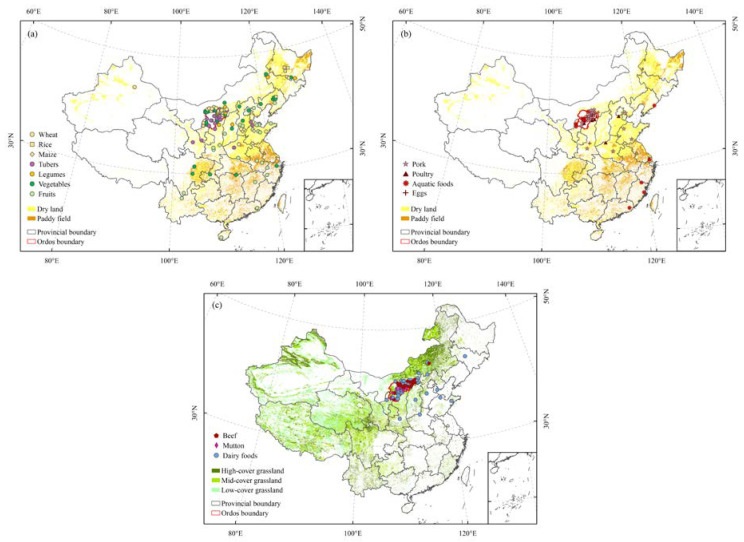
The production areas of plant-based foods (**a**) and animal-based foods (**b**) distributed in cropland and animal-based foods (**c**) distributed in grassland.

By analyzing 359 food samples from the Ordos Market Supervision Administration, we found that residents’ food sources involved 25 provinces, municipalities, and autonomous regions in China (Figure 6, Table 4).

Food consumption of residents especially depended on the production of local livestock products and local leading agricultural products. As one of the major maize-producing areas in China, Ordos was highly dependent on local maize production. Since 2013, the self-sufficiency rate of maize has been maintained at about 2000%. As well as being used for local consumption, maize was also sold to Hunan, Hubei, Chongqing, and other provinces. However, because the edible maize produced locally account for less than 20% of the total maize, and the rest was feed maize [32], Ordos still needed to purchase maize from other major maize-producing areas, such as Hebei Province and Shandong Province (Figure 6a, Table 4). Potatoes consumed by residents were also mainly from local production, with the annual yield (10 × 10^4^ t) exceeding annual consumption (2 × 10^4^ t) by five times. Ordos residents also liked to eat high-quality potato products from Dingxi City, Gansu Province (the capital of Chinese potato production) and Guyuan City, Ningxia Hui Autonomous Region. Pork consumption became less dependent on local production, and pork was purchased from neighboring provinces, such as Hebei and Shaanxi, to meet consumer demand (Figure 6b, Table 4). Livestock products were highly dependent on local production (Figure 6c, Table 4), especially mutton, such as Albas goat meat of Otok Banner and Tarian Gol goat meat of Hangjin Banner. Ordos residents preferred dairy foods, such as milk, yogurt, cheese and kumiss, reflecting the dietary habits of typical pastoral areas. The main sources of dairy foods included Ordos, Hohhot (the capital of Chinese dairy) and Ulanqab, where there are high-quality pastures, but some dairy foods were bought from nearby provinces, such as Hebei, Shanxi, and Shaanxi, due to the high prices of local dairy foods.

The foods that depended on non-local production included some agricultural and fishery products, which had obvious distribution differences in the supply places and were mainly distributed in the agricultural areas of central and eastern China (Figure 6a,b). The supply places of agricultural products were concentrated in Inner Mongolia and its neighboring provinces, while the fishery products were scattered in the coastal provinces. The production capacities of wheat and rice in the pastoral area were insufficient, and their annual average self-sufficiency rates were 32.77% and 6.88%, respectively. Ordos relied on purchasing more than 20 × 10^4^ t of wheat and rice from other places annually to balance local supply and demand. The major supply area of wheat was Hetao Plain in Bayannur, Inner Mongolia, which has a specialty named Wuyuan wheat. The supply areas also included areas on the North China Plain, such as Xingtai of Hebei Province, Zhengzhou of Henan Province, and Tsingtao of Shandong Province, as well as the wheat-producing areas of Changji prefecture of Xinjiang Uyghur Autonomous Region (Figure 6a, Table 4). The rice was mainly purchased from Northeast China (such as Wuchang rice in Harbin, Heilongjiang Province), followed by Shizuishan, Ningxia Hui Autonomous Region. It is worth noting that although the self-sufficiency rates of vegetables and fruits were both higher than 100%, they were heavily dependent on non-local production. The supply areas of vegetables were scattered in 10 provinces and fruits in 14 provinces. This was because the growth of vegetables and fruits was greatly affected by temperature, precipitation, light, and soil, so the production has regional and seasonal characteristics. The locality mainly produced summer and autumn vegetables, such as capsicum, brassica campestris, and brassica juncea. The non-local vegetables included cabbage and pickled cabbage, mainly from Baotou and Bayannur of Inner Mongolia. The non-local fruits included crown pear in Zhaoxian, Hebei Province, Kyoho grape in Weifang, Shandong Province, Xuxiang kiwifruit in Zhouzhi, Shaanxi Province, and red Pitaya fruit in Hainan Province. The external dependencies of poultry and eggs were also increasing. The poultry was imported from Inner Mongolia, and the Hebei and Henan provinces, and the eggs were imported mainly from Shaanxi Province (Figure 6b, Table 4). Finally, Ordos has developed aquaculture along the Yellow River, and the self-sufficiency rate of aquatic foods was higher than 100%. However, there were few aquatic species locally, mainly including Otok spirulina and Batu Bay carp. The supply of other aquatic foods was insufficient, relying on imports from eastern coastal provinces, such as Liaoning, Shanghai, Zhejiang, Fujian, and Guangdong. In addition, Ningxia grass carp in western China was also very popular in Ordos.

## 4. Discussion

The situation of food self-sufficiency was not optimistic in Ordos. In 2019, a designated supervision site at the airport was built in Ordos, where imported fruits and aquatic foods can be directly transported from abroad. This has deepened Ordos’s dependence on international agricultural product markets, while weakening local production. Given the uncertainties of the international food supply chain during the COVID-19 pandemic, it is time to strengthen local food production to ensure food security [33].

To achieve food security, the first step is to reduce dependence on non-local food and optimize local production. Constraints on local production include less cultivated land, insufficient fresh water, and the deterioration of soil quality [34]. There are few cultivated land resources in Ordos pastoral areas, so it is not a long-term solution to increase the crop yield continuously by expanding the sown area. Instead, it is necessary to ameliorate soil and improve crop varieties [35] through ecological and agricultural technologies, and to adjust the structure of the agricultural and animal husbandry industries to achieve optimal production. A study established an optimization model of crop farming structure based on multi-objective genetic algorithms, and found that after optimization, the sown area of maize in Ordos should be reduced by 40.5%, while the sown area of wheat, potatoes, legumes, and vegetables should be increased by 1.1%, 18.3%, 16.5% and 4.2%, respectively [31]. While adjusting the planting structure of crops, it is also important to ensure that local products are given priority for the needs of local residents. However, currently, native wheat in Ordos is mainly used for industrial production [36], native maize is mainly used as feed [32], and 85% of vegetables are exported to Guangdong, Hebei, Shandong, Beijing, Tianjin, and other provinces [37].

Consumers’ preference for local food also contributes to food safety. A total of 91.58% of the mutton consumed by shepherd households was produced by their own households, and 45.13% of cattle-herding households consumed their own produce [36]. However, the local supply chain is economically feasible only if consumers are willing to pay higher prices for intermediate consumption invested in the local production process [38]. A study of German consumers showed a high demand for animal-derived foods made from livestock fed with local feed [39]. Another survey of American college students showed that young consumers were more likely to consume locally produced food [40]. These results may be explained by the fact that foods produced by local producers conformed more closely to the dietary habits of local residents than those produced by “regional” or “national” foods [41]. We noted that Ordos has a strong dairy production capacity, but per capita consumption has been at a low level. One of the important reasons is that the Chinese have a high incidence of lactase deficiency, resulting in gastrointestinal discomfort symptoms after drinking milk [42]. The consumption behavior of dairy foods has obvious household characteristics; that is, whether there are elderly people and children in the households has a significant impact on consumption [43]. In a word, the low per capita consumption of dairy foods, whether in Ordos or nationwide, is due to the combined influence of demographic structure, income level, dairy prices, consumption habits, and dietary preferences.

For food supply, establishing a shorter, structured supply chain can save transportation time, reduce transportation costs, and enable residents to obtain fresh food [44]. For food consumption, improving the capacity of food self-sufficiency needs to change consumer behaviors, such as forming the habit of eating local and seasonal food and reducing food waste [45]. Ensuring food safety is a long-term systematic project, which needs to focus on all aspects of the food system, including production, processing, storage, circulation, and consumption [46], as well as strengthening the food safety management throughout the whole industrial chain [47,48].

## 5. Conclusions

Exploring the characteristics and relationships between food production and consumption in pastoral areas is helpful to understand the food security situation of local residents. We systematically evaluated the production, consumption, and self-sufficiency rates of different food types in Ordos during 2000–2020, and analyzed the local dependence of food consumption.

The food production and consumption structures of Ordos were both dominated by grains, accounting for more than 50% of the total. The total food production showed a growth trend except for wheat and potatoes and total consumption also increased except for potatoes. The consumption structure was still unreasonable, reflecting an excessive consumption of grains and meat, and insufficient consumption of vegetables, fruits, and dairy foods.

The self-sufficiency rates of total food ranged from 228.13% to 547.93%, which seems to imply that the population have achieved self-sufficiency. However, five types of agricultural products, namely wheat, rice, pork, poultry, and eggs, have not achieved self-sufficiency. Meanwhile, with the increase in consumer demand, the self-sufficiency rate of meat, eggs, and dairy foods has gradually declined.

We also found that livestock products consumed by residents were highly dependent on local production, and agricultural and fishery products were more dependent on external supply from other regions. The food consumption of residents was affected by cultivation structure and dietary preference, so the realization of food security could consider the following two aspects: optimizing local production and improving the competitiveness of local food products.

## Figures and Tables

**Table 1:** Physical conditions and social–economic conditions in Ordos.

**Physical Conditions**	**Land Area** **(km^2^)**	**Elevation** **(m)**	**Average** **Annual** **Temperature** **(℃)**	**Average** **Annual** **Precipitation** **(mm)**	**Average** **Annual** **Evaporation** **(mm)**	**Cropland** **(×10^3^ ha; %)**	**Grassland** **(×10^3^ ha; %)**
	86,882	1000–1500	6.2	348	2506	435.8; 5.1	5138.2; 60.1
**Socio-economic conditions in 2020**	**Permanent population** **(×10^3^)**	**Population of Han and Mongolian** **nationalities** **(×10^3^)**	**Urbanization** **Rate** **(%)**	**Output value of agriculture and animal husbandry** **(×10^9^ Yuan)**	**Disposable** **income per capita of urban and rural residents** **(Yuan)**	**Disposable** **income per capita of agriculture and animal husbandry (Yuan)**	**Consumption expenditure per capita of urban and rural residents** **(Yuan)**
	2155.6	1924; 208	77.45	13.24; 8.48	50,300; 21,600	7149; 3988	29,002; 16,206

**Table 2:** Survey sample sizes of rural and pastoral residents during 2000–2015.

Year	2000	2001	2002	2003	2004	2005	2006	2007	2008	2009	2010	2011	2012	2013	2014	2015
Number of households surveyed	295	300	300	300	300	300	300	300	300	300	275	420	420	382	381	381
Number of respondents	1161	1186	1191	1102	1097	1078	1055	1026	1035	972	872	1180	1186	1066	1067	1105

**Table 3:** The main food types of residents in Ordos.

Food Types	Specific Description
Grains	Wheat, rice, maize, other grains (millet, sorghum, highland barley, naked oats, broom corn millet, buckwheat, barley, etc.)
Tubers	Sweet potato, potato, other tubers (dioscorea, purple potato, cassava, taro, etc.)
Legumes	Soybean, other legumes (red adzuki bean, mung bean, etc.)
Vegetables	Melon vegetables, solanaceae vegetables, cabbages, leafy greens
Fruits	Muskmelon, watermelon, apple, pear, grape
Meat and eggs	Livestock meat: pork, beef, mutton
	Poultry: chicken, duck, goose
	Aquatic foods: fish, algae
	Eggs: fresh eggs, egg products
Dairy foods	Milk, yogurt, dried milk

**Table 4:** Distribution of food production areas in 25 provinces. The values represent the frequency of food production areas in different provinces among the 359 food samples sampled by Ordos Market Supervision Administration.

Production Areas	Grains			Tubers	Legumes	Vegetables	Fruits	Meat and Eggs						Dairy Foods	Total
	Wheat	Rice	Maize					Pork	Beef	Mutton	Poultry	Aquatic Foods	Eggs		
1. Local															
Ordos		2	2	24	5	18	4	25	21	6	1	13	5	37	163
2. Non-local															
Inner Mongolia (excepting Ordos)	11		1	2	2	20	2	3	13		2	1	1	24	82
Hebei	1		2	1		2	5	1			1			2	15
Shaanxi				2	2		3	1					4	1	13
Shandong	1		1	1				4						2	9
Henan	1			1			1	3			1			1	8
Heilongjiang		6			1	1									8
Liaoning		2				4	1					1			8
Shanxi					2	1	1							4	8
Zhejiang						1	5					1			7
Ningxia		2		1								2		1	6
Jiangxi							6								6
Guangdong							3					1			4
Sichuan						2	1								3
Jilin					1									1	2
Shanghai					1							1			2
Hunan						2									2
Anhui							2								2
Fujian							1					1			2
Hainan							2								2
Tianjin														2	2
Xinjiang	1														1
Gansu				1											1
Beijing						1									1
Chongqing						1									1
Yunnan							1								1
Total	15	12	6	33	14	53	38	37	34	6	5	21	10	75	359

## Data Availability

Data are contained within the article.

## Supplementary Materials

The following supporting information can be downloaded at: https://www.mdpi.com/article/10.3390/foods12051066/s1, Questionnaire for Food Production and Consumption of Farmers and Herdsmen in Inner Mongolia.
