# Supplementary material for: Food Production and Consumption in Ordos of Inner Mongolia
Source: Foods. 2023 Mar 2;12(5):1066. doi: 10.3390/foods12051066 (PMC10000956; doi:10.3390/foods12051066)
Supplement: Supplementary file 1 [file foods-12-01066-s001.zip › foods-2177263-supplementary.pdf]

Questionnaire for Food Production and Consumption of Farmers and Herdsmen in Inner Mongolia

Questionnaire Number_______ **Investigator**______________ **Date** ______(mm) ______(dd) ______(yyyy)

Survey location: Village_________ Town_________ County (Banner) _________ City (League) ________

Contact: Person to contact______________________________ Telephone______________________________

1. The basic information of your family members

**2018**	Gender [1]	Age	Nationality [2]	Degree of education [3]	Main occupation [4]	Part-time occupation [4]	Settlement [5]	Reasons for living out [6]	Time spent living out
Yourself									
Spouse									
Mother									
Father									
Children									
Other members									
**10 years ago**	Household size: _________person				Occupation: _____herdsmen, _____farmers, _____workers, _____institution staff, _____students, _____others				
**20 years ago**	Household size: _________person				Occupation: _____herdsmen, _____farmers, _____workers, _____institution staff, _____students, _____others				

[1] Gender: 1—male, 2—female

[2] Nationality: 1—Mongolian, 2— Han nationality, 3—other (please state)

[3] Degree of education: 1—illiteracy, 2—primary school, 3—junior school, 4—high school, 5—bachelor, 6—master, 7—doctor

[4] Occupation: 1—herdsman, 2—farmer, 3—worker, 4—institution staff, 5—student, 6—others (please state)

[5] Settlement: 1—local village (town), 2—local county (banner), 3—local city (league), 4—local province, 5—other provinces (please state)

[6] Reasons for living out: 1—work, 2—marriage, 3—study, 4—other (please state)

2. Household income

**2018 (CNY)**	Grazing	Breeding	Planting	Manual work	Subsidy	Other	Total	Deposit
**10 years ago**	Change: 1—increase, 2—decrease, 3—no change							
	Reasons for change: 1—household size, 2—manual work, 3—subsidy, 4—environmental protection, 5—others (please state)							
**20 years ago**	Change: 1—increase, 2—decrease, 3—no change							
	Reasons for change: 1—household size, 2—manual work, 3—subsidy, 4—environmental protection, 5—others (please state)							

3. Household food expenditure

2018 (CNY)	Grain	Tubers	Legumes	Vegetables	Fruits	Meat	Eggs	Dairy foods	Total
**10 years ago**	Change: 1—increase, 2—decrease, 3—no change								
	Reasons for change: 1—household size, 2—income, 3—consumption habit, 4—others (please state)								
**20 years ago**	Change: 1—increase, 2—decrease, 3—no change								
	Reasons for change: 1—household size, 2—income, 3—consumption habit, 4—others (please state)								

4. Land use

Period	Area (ha)	Uncultivated (ha)	Revert cultivated land/ Pasture (ha)	Livestock capacity (ha/100 sheep)	Reasons for change*
**Grassland**					
2018	Total:<br>Summer:<br>Winter:		Area (ha): ___________<br>Subsidy (CNY): ___________		
10 years ago	Total:<br>Summer:<br>Winter:		Area (ha): ___________<br>Subsidy (CNY): ___________		
20 years ago	Total:<br>Summer:<br>Winter:		Area (ha): ___________<br>Subsidy (CNY): ___________		
**Cropland**					
2018			Area (ha): ___________<br>Subsidy (CNY): ___________	—	
10 years ago			Area (ha): ___________<br>Subsidy (CNY): ___________	—	
20 years ago			Area (ha): ___________<br>Subsidy (CNY): ___________	—	

* Reason: 1—income, 2—labor force, 3—government subsidy, 4—environmental protection, 5—others (please state)

5. Household production and consumption of animal-based foods

2018	Breeding (amount)			Breeding mode (captive/ free-range)	Self-consumption (kg)	Sell		Purchase		
	Livestock on hand	Slaughter	Disease / disaster loss			Quantity (kg)	Unit price (CNY)	Quantity (kg)	Unit price (CNY)	Place of origin
Pig										
Sheep										
Cattle										
Poultry										
Fish										
Eggs	Production (kg):									

Dairy foods	Production (kg):									
Others										
**Consumption change**	1—increase, 2—decrease, 3—no change									
**Reasons for change**	1—income, 2—price 3—place of origin, 4—consumption habit, 5—grassland area, 6—others (please state)									
**10 years ago**	Breeding (amount)			Breeding mode (captive/ free-range)	Self-consumption (kg)	Sell		Purchase		
	Livestock on hand	Slaughter	Disease / disaster loss			Quantity(kg)	Unit price (CNY)	Quantity (kg)	Unit price (CNY)	Place of origin
Pig										
Sheep										
Cattle										
Poultry										
Fish										
Eggs	Production (kg):									
Dairy foods	Production (kg):									
Others										
**Consumption change**	1—increase, 2—decrease, 3—no change									
**Reasons for change**	1—income, 2—price 3—place of origin, 4—consumption habit, 5—grassland area, 6—others (please state)									
**20 years ago**	Breeding (amount)			Breeding mode (captive/ free-range)	Self-consumption (kg)	Sell		Purchase		
	Livestock on hand	Slaughter	Disease / disaster loss			Quantity (kg)	Unit price (CNY)	Quantity (kg)	Unit price (CNY)	Place of origin
Pig										
Sheep										
Cattle										
Poultry										
Fish										
Eggs	Production (kg):									
Dairy foods	Production (kg):									
Others										

6. Household production and consumption of plant-based foods

**2018**	Planting		Self-consumption (kg)	Sell		Purchase		
	Area (ha)	Production (kg)		Quantity (kg)	Unit price (CNY)	Quantity (kg)	Unit price (CNY)	Place of origin
Wheat								
Rice								

Maize								
Potato								
Soybean								
Vegetables								
Fruits								
Others								
**Consumption change**	1—increase, 2—decrease, 3—no change							
**Reasons for change**	1—income, 2—price 3—place of origin, 4—consumption habit, 5—cropland area, 6—others (please state)							
**10 years ago**	Planting		Self-consumption (kg)	Sell		Purchase		
	Area (ha)	Production (kg)		Quantity (kg)	Unit price (CNY)	Quantity (kg)	Unit price (CNY)	Place of origin
Wheat								
Rice								
Maize								
Potato								
Soybean								
Vegetables								
Fruits								
Others								
**Consumption change**	1—increase, 2—decrease, 3—no change							
**Reasons for change**	1—income, 2—price 3—place of origin, 4—consumption habit, 5—cropland area, 6—others (please state)							
**20 years ago**	Planting		Self-consumption (kg)	Sell		Purchase		
	Area (ha)	Production (kg)		Quantity (kg)	Unit price(CNY)	Quantity (kg)	Unit price (CNY)	Place of origin
Wheat								
Rice								
Maize								
Potato								
Soybean								
Vegetables								
Fruits								
Others								
